# Frailty predicts all-cause and cause-specific mortality among older adults in Austria: 8-year mortality follow-up of the Austrian Health Interview Survey (ATHIS 2014)

**DOI:** 10.1186/s12877-023-04633-3

**Published:** 2024-01-03

**Authors:** Erwin Stolz, Anna Schultz, Sandra Schüssler, Hannes Mayerl, Emiel O. Hoogendijk, Wolfgang Freidl

**Affiliations:** 1 Institute of Social Medicine and Epidemiology, Medical University of Graz, Graz, Austria; 2 Department of Epidemiology & Data Science, Amsterdam Public Health Research Institute, Amsterdam UMC-Location VU University Medical Center, Amsterdam, the Netherlands

**Keywords:** Frailty index, All-cause mortality, Cause-specific mortality, CVD, Cancer

## Abstract

**Background:**

The frailty index (FI) is an established predictor of all-cause mortality among older adults, but less is known with regard to cause-specific mortality, and whether the predictive power of the FI varies between men and women and by socio-economic position.

**Methods:**

We assessed all-cause and cause-specific mortality during 8 years of follow-up (median = 7 years) among the population-representative sample of older adults (65 + , *n* = 2,561) from the European Health Interview Survey in Austria (ATHIS 2014). A FI at baseline was constructed from 41 health deficits. Official cause of death information from Statistics Austria was linked with the survey data by the Austrian Micro Data Center (AMDC). Next to all-cause mortality, we differentiated between mortality from cardiovascular diseases (CVD), cancer, and other causes. Cox proportional hazard models adjusted for socio-demographic variables and causes of death as competing risks were used to assess mortality prediction.

**Results:**

Among the participants, 43.5% were robust (FI < 0.10), 37.7% pre-frail (FI = 0.10–0.21), and 18.7% were frail (FI > 0.21). 405 (15.8%) participants died during follow-up. Among the deceased, 148 (36.5%) died from CVD, 127 (31.4%) died from cancer, and 130 (32.1%) died from other causes of death. The FI predicted all-cause (hazard ratio, HR = 1.33 per 0.1 FI and HR = 2.4 for frail compared to robust older adults) and cause-specific mortality risk (HR_CVD_ = 1.25/2.46, HR_cancer_ = 1.19/1.47, HR_other_ = 1.49/3.59). Area under the curve (AUC) values were acceptable for CVD mortality (0.78) and other causes of death (0.74), and poor for cancer mortality (0.64).

**Conclusions:**

The FI predicts all-cause and cause-specific mortality (CVD, other causes) well, which points to its relevance as a potential screening tool for risk stratification among community-dwelling older adults.

**Supplementary Information:**

The online version contains supplementary material available at 10.1186/s12877-023-04633-3.

## Background

Frailty is the result of a cumulative decline in multiple physiological systems and is defined as a state of increased vulnerability among older adults with regard to adverse outcomes [1]. Frailty has major implications for clinical practice and public health [2] and can help identifying older individuals at a high risk of health deterioration and mortality, guiding targeted care efforts [3]. One of the two main operationalizations of frailty [4] is the well established cumulative deficit model of frailty as outlined by Rockwood and Mitnitski [5–7], which depicts frailty as a state of risk due to a large number of health deficits summarized in a continuous frailty index (FI). A systematic review and meta-analysis [8] of 19 studies showed that baseline FI differences consistently predicted all-cause mortality among community-dwelling older adults, and a recent systematic review [9] reported that in 6 out of 8 (75%) selected studies, the continuous FI had acceptable discriminatory power (area-under-the-curve, AUC, > 0.70) for mortality prediction. In addition to one-time assessments, recent studies [10–13] also suggest that FI changes predict mortality above and beyond baseline differences.

In contrast to the established link between the FI and all-cause mortality, less is known about the FI’s relation with cause-specific mortality [14], although this might provide insights about the mechanisms linking frailty and mortality. While the FI has been suggested as a tool for risk stratification in various patient sub-populations based on its predictive capacity [2], there is limited evidence [13, 15–19] whether the FI also predicts cause-specific mortality, i.e. whether frail older adults are more likely to die of some underlying causes of death than others. The few studies available to date indicate that the FI is better at predicting respiratory and cardiovascular disease (CVD)-related mortality compared to deaths from cancer, although some of these patterns may be gender-specific [15]. Assessing whether the vulnerability of accumulated health problems described by the FI varies by the type of underlying disease that later causes death could inform risk stratification and prevention efforts. Furthermore, it is relevant to ascertain whether the FI predicts overall and cause-specific mortality equally well for sub-populations with known FI differentials, that is, for example between men and women [20] and between people with high- and low socio-economic position [21].

In this study, we aim to assess both all-cause and cause-specific mortality risk by the level of frailty among a large population-representative sample of community-dwelling older adults in Austria. Our expectation is that compared to fit older adults, those with more health problems would be more likely (1) to die from any cause of death, and (2) to die from CVD compared to cancer, as the latter may, depending on the body site, prove terminal even among fairly robust older individuals.

## Methods

### Data

Our study is based on data from the population-representative Austrian Health Information Survey (ATHIS) 2014. As part of the second wave of the European Health Interview Survey (EHIS) [22] and conducted by Statistics Austria, 38,768 community-dwelling individuals aged 15 years and above were randomly selected based on the central population register and stratified by geographic region for participation. Of the approached individuals, 15,771 (response rate = 40.7%) agreed to participate, and 2,561 were aged 65 years and over, constituting the analytical sample for this study. Telephone-based interviews in the ATHIS 2014 were conducted between October 2013 and June 2015. Participation in the study was voluntary, and all participants were informed about the aims and contents of the study as well as about data protection. Respondents provided informed consent before the telephone interview.

### Frailty assessment

Frailty was operationalized with the health deficit accumulation approach, and calculated following standard procedure [23] from 41 age-related health deficits which covered multiple physiological systems and included chronic diseases, limitations in basic and instrumental activities of daily living, mobility and sensory impairment, poor self-rated health, somatic and psychological symptoms, body mass deficits and limited physical activity (Table 1). All health deficits had 5% or less missing data and the FI was calculated for all participants who had more than 80% valid data, which included all respondents. The FI was calculated as the number of health deficits of each person divided by the number of (valid) health deficits considered so that, for example, an older adult with 10 out of 41 health deficits obtained an FI of 5/41 = 0.12. In addition to the continuous FI, we also defined three broad frailty categories: participants with a FI < 0.1 were considered ‘robust’, those between 0.1 and 0.21 as ‘pre-frail’, and those above 0.21 as ‘frail’ [24].

**Table 1:** Health deficits of the frailty index (FI)

Health deficit	Coding	Prevalence (%)	Missings n (%)
Self-rated health	very good = 0,	0 = 18.1	-
	good = 0.25,	0.25 = 42.9	
	moderate = 0.50,	0.50 = 31.1	
	poor = 0.75,	0.75 = 6.2	
	very poor = 1	1 = 1.7	
Chronic disease	no = 0, yes = 1	1 = 53.6	-
Global activity limitation indicator (GALI)	not limited = 0,	0 = 52.6	-
	somewhat limited = 0.50	0.50 = 35.3	
	stongly limited = 1	1 = 12.1	
Asthma	no = 0, yes = 1	1 = 5.6	-
Chronic obstructive pulmonary disease	no = 0, yes = 1	1 = 8.6	-
Myocardial infarction^a^	no = 0, yes = 1	1 = 2.9	-
Coronary heart disease/angina pectoris^a^	no = 0, yes = 1	1 = 6.2	-
Hypertension^a^	no = 0, yes = 1	1 = 46.6	-
Stroke^a^	no = 0, yes = 1	1 = 2.2	-
Arthritis	no = 0, yes = 1	1 = 30.0	-
Upper back pain	no = 0, yes = 1	1 = 25.6	-
Lower back pain	no = 0, yes = 1	1 = 35.5	-
Diabetes	no = 0, yes = 1	1 = 1.9	-
Incontinency	no = 0, yes = 1	1 = 10.3	-
Renal disease	no = 0, yes = 1	1 = 3.4	-
Depression	no = 0, yes = 1	1 = 9.2	-
Chronic headache	no = 0, yes = 1	1 = 4.2	-
Gastroenteritis	no = 0, yes = 1	1 = 2.3	-
Vision problems	none = 0	0 = 85.4	5 (0.1)
	some = 0.33	0.33 = 12.7	
	substantial = 0.66	0.66 = 1.8	
	almost blind/blind = 1	1 = 0.1	
Hearing problems in silent room	none = 0	0 = 85.4	5 (0.1)
	some = 0.33	0.33 = 12.8	
	substantial = 0.66	0.66 = 1.6	
	almost deaf/deaf = 1	1 = 0.1	
Hearing problems in loud room	none = 0	0 = 55.6	9 (0.3)
	some = 0.33	0.33 = 36.2	
	substantial = 0.66	0.66 = 8.0	
	almost deaf/deaf = 1	1 = 0.1	
Difficulty walking 500 m	none = 0	0 = 83.3	-
	some = 0.33	0.33 = 8.2	
	substantial = 0.66	0.66 = 5.5	
	cannot do it = 1	1 = 0.3	
Difficulty walking flight of stairs	none = 0	0 = 80.0	-
	some = 0.33	0.33 = 12.8	
	substantial = 0.66	0.66 = 5.5	
	cannot do it = 1	1 = 0.2	
Difficulty eating	none = 0	0 = 98.8	-
	some = 0.33	0.33 = 0.1	
	substantial = 0.66	0.66 = 0.0	
	cannot do it = 1	1 = 0.0	
Difficulty sitting down and standing up	none = 0	0 = 91.6	-
	some = 0.33	0.33 = 6.9	
	substantial = 0.66	0.66 = 1.3	
	cannot do it = 1	1 = 0.0	
Difficulty dressing	none = 0	0 = 94.0	-
	some = 0.33	0.33 = 4.6	
	substantial = 0.66	0.66 = 1.1	
	cannot do it = 1	1 = 0.0	
Difficulty using toilet	none = 0	0 = 97.8	-
	some = 0.33	0.33 = 1.4	
	substantial = 0.66	0.66 = 0.1	
	cannot do it = 1	1 = 0.01	
Difficulty bathing/showering	none = 0	0 = 93.5	-
	some = 0.33	0.33 = 4.1	
	substantial = 0.66	0.66 = 1.4	
	cannot do it = 1	1 = 0.1	
Difficulty using phone	none = 0	0 = 98.1	4 (0.2)
	some = 0.33	0.33 = 0.9	
	substantial = 0.66	0.66 = 0.7	
	cannot do it = 1	1 = 0.0	
Difficulty shopping groceries	none = 0	0 = 92.0	92 (3.5)
	some = 0.33	0.33 = 4.0	
	substantial = 0.66	0.66 = 1.4	
	cannot do it = 1	1 = 2.8	
Difficulty taking medicine	none = 0	0 = 0.98	145 (5.7)
	some = 0.33	0.33 = 0.7	
	substantial = 0.66	0.66 = 0.5	
	cannot do it = 1	1 = 0.7	
Difficulty doing light housework	none = 0	0 = 94.8	125 (4.9)
	some = 0.33	0.33 = 3.0	
	substantial = 0.66	0.66 = 0.7	
	cannot do it = 1	1 = 0.1	
Difficulty managing finances	none = 0	0 = 96.4	111 (4.3)
	some = 0.33	0.33 = 1.6	
	substantial = 0.66	0.66 = 0.6	
	cannot do it = 1	1 = 1.4	
Pain level	none/almost none = 0	0 = 44.9	-
	light = 0.25	0.25 = 21.3	
	moderate = 0.50	0.50 = 20.7	
	severe = 0.75	0.75 = 8.8	
	very severe = 1	1 = 4.3	
How often sad, depressed or hopeless	0 = never	0 = 79.8	-
	0.33 = some days	0.33 = 17.6	
	0.66 = more often than not	0.66 = 1.3	
	1 = almost every day	1 = 1.3	
How often sleep problems	0 = never	0 = 53.8	-
	0.33 = some days	0.33 = 27.7	
	0.66 = more often than not	0.66 = 5.1	
	1 = almost every day	1 = 13.4	
How often do you feel tired or weak	0 = never	0 = 53.0	-
	0.33 = some days	0.33 = 37.4	
	0.66 = more often than not	0.66 = 4.5	
	1 = almost every day	1 = 5.0	
How often do you have little/a lot of appetite	0 = never	0 = 89.8	-
	0.33 = some days	0.33 = 7.3	
	0.66 = more often than not	0.66 = 10.5	
	1 = almost every day	1 = 1.8	
How often do you have difficulties concentrating	0 = never	0 = 87.2	-
	0.33 = some days	0.33 = 10.3	
	0.66 = more often than not	0.66 = 11.3	
	1 = almost every day	1 = 1.3	
Physical activity	0 = regularly 1 = never or less often than once per week	1 = 31.8	-
BMI deficit	BMI > = 18.5 & BMI < 25 = 0	0 = 37.5	-
	BMI > = 25 & BMI < = 30 = 0.50	0.50 = 42.7	
	BMI < 18.5 or BMI > 30 = 1	1 = 19.8	

^a^Excluded from FI in sensitivity analysis

Relevant socio-demographic variables included age (in years), sex (male/female), education (low = ISCED11 level 1–2, medium = level 3–4, high = level 5–8), and living alone (no/yes).

### Mortality ascertainment

Information on the vital status of ATHIS participants up to 31.12.2021 came from official cause-of-death statistics collected by Statistics Austria and were matched with ATHIS survey data for this analysis by the Austrian Micro Data Center (AMDC). Matching was 100% complete, as was mortality follow-up data. In Austria, certification of the underlying cause of death is conducted by coroners, pathologists, or forensic pathologists following the International Classification of Diseases, 10th version (ICD-10). Causes of death were classified as either circulatory diseases (ICD-10: I00-99), cancer (ICD-10: C00-99), or other cause of death. Additional groups of causes of death or specific diseases within CVD or cancer could not be analysed in detail due to their small numbers. Exact interview and death dates (per day) were available and time between the ATHIS 2014 survey interview and date of death or the end of follow-up (31.12.2021) respectively, was calculated in years. Median mortality follow-up duration across all ATHIS participants was 7.2 (interquartile range, IQR, = 0.9) years.

### Statistical analysis

We provide descriptive statistics and plots to characterise the FI instrument in ATHIS 2014. Kaplan–Meier plots were used to describe all-cause mortality risk over time, and cumulative incidences are shown for cause-specific mortality risk by FI categories. Cox proportional hazard models adjusted for socio-demographic variables were used to estimate the hazard ratios (HR) per 0.1 FI increment as well as by FI categories (robust/pre-frail/frail) for all-cause and cause-specific mortality. For the latter, we adjusted for causes of death as competing risks by using the cause-specific hazard model, i.e. participants who experienced death from one cause of death were treated as censored at the time of the occurrence of death. Furthermore, we conducted a sensitivity analysis for CVD mortality, where we excluded CVD diagnoses and risk factors (chronic heart disease, heart infarction, stroke, and hypertension) from the calculation of the FI. Reported AUC values refer to Harrell’s C-index/concordance-index. The proportional hazards assumption was tested (and supported) by scaled Schoenfeld residuals. We tested for interaction effects of the FI with sex and level of education by assessing model fit changes (likelihood ratio test). All statistical analyses were conducted with R (4.1.3).

## Results

**Fig. 1:**
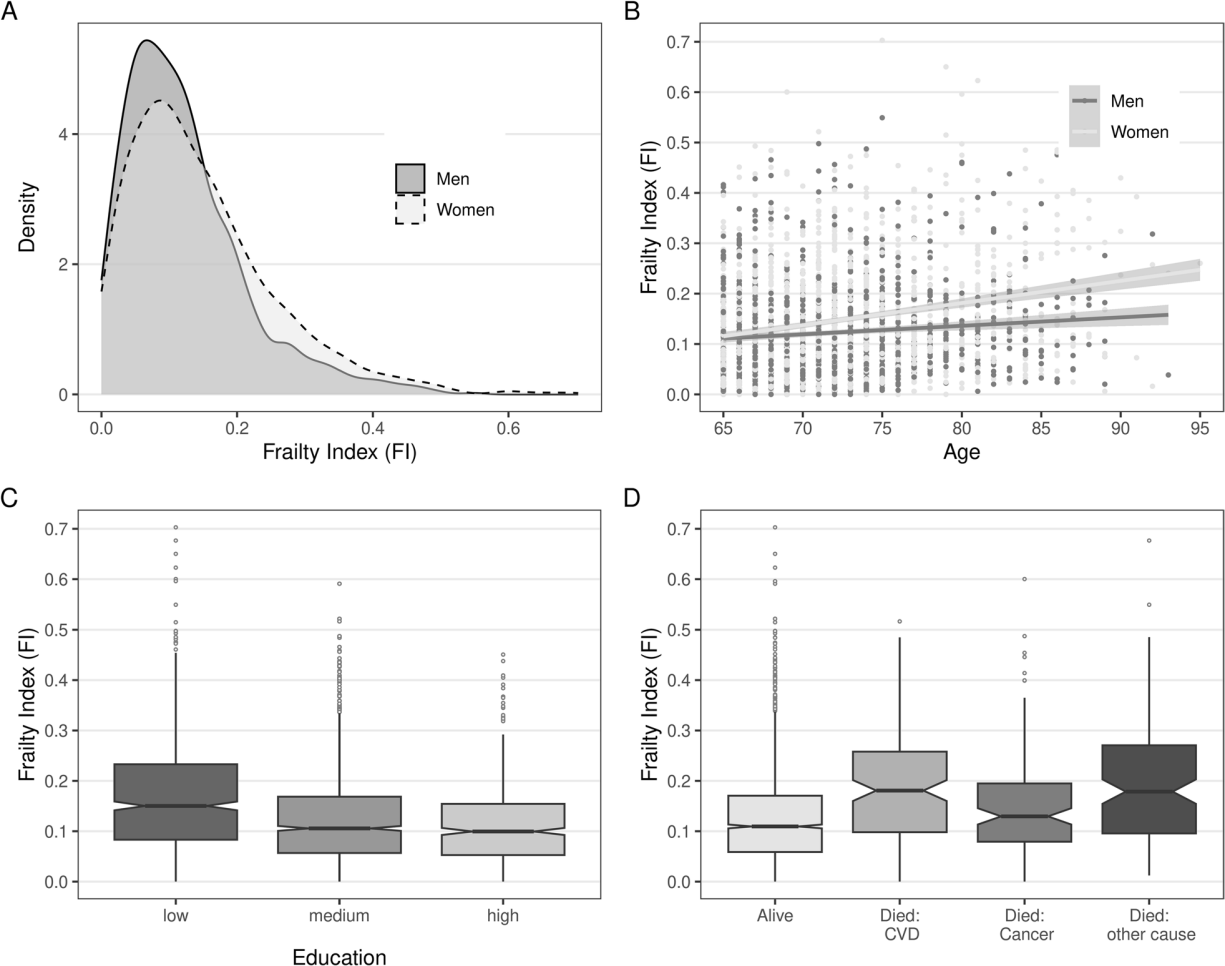
Descriptive statistics of the frailty index (FI)

Women constituted 54.2% of the sample, 28.3% of the participants completed primary schooling, 51.3% finished secondary education, and 20.4% completed higher education, and the average age at baseline was 72.1 (SD = 5.8) years. Additional sample characteristics of the participants stratified by FI category are presented in Table 2. The mean/median FI level was 0.14/0.11 (SD = 0.10, IQR = 0.12) and FI values ranged between 0–0.70. Sub-maximum FI values were 0.34 (95th percentile) respectively 0.46 (99th percentile). Plots A, B, and C in Fig. 1 show that the distribution of the FI was right-skewed, that the FI increased with age, was higher among women than men, and higher among those with lower education. According to the cut-off values, 43.5% of the participants were robust (FI < 0.10), 37.7% pre-frail (FI 0.10–0.21), and 18.7% frail (FI > 0.21, that is, 9 or more health deficits out of 41).

**Table 2:** Sample characteristics, total and by frailty index (FI) category

	Total	Robust (FI < 0.1)	Pre-frail (FI 0.10–0.21)	Frail (FI > 0.21)
	*N* = 2,561	*N* = 1,115	*N* = 967	*n* = 426
Status				
Alive/censored	2,156 (84.2%)	994 (89.1%)	816 (84.3%)	306 (71.8%)
Deceased	405 (15.8%)	121 (10.9%)	151 (15.6%)	133 (27.8%)
CVD	148 (5.8%)	40 (3.6%)	54 (5.6%)	54 (11.3%)
Cancer	127 (5.0%)	48 (4.3%)	51 (5.3%)	28 (5.8%)
Other	130 (5.1%)	33 (3.0%)	46 (4.8%)	51 (10.6%)
Female sex	1400 (54.7%)	557 (50.0%)	521 (53.9%)	322 (67.2%)
Education: low	726 (28.3%)	234 (21.0%)	266 (27.5%)	226 (47.2%)
Education: medium	1,313 (51.3%)	619 (55.5%)	498 (51.5%)	196 (40.9%)
Education: high	522 (20.4%)	262 (23.5%)	203 (21.0%)	57 (11.9%)
Age: mean (SD) in years	72.1 (5.8)	71.2 (5.4)	72.5 (5.7)	73.7 (6.6)
Follow-up total sample: median (IQR) in years	7.2 (0.9)	7.2 (0.9)	7.2 (0.9)	7.2 (1.1)
Follow-up deceased: median (IQR) in years	4.7 (3.4)	4.7 (3.0)	4.9 (3.1)	4.2 (3.3)

From the 2,561 participants recruited between 2013–2015, who were followed up for a median of 7.2 (IQR = 0.9) years (total follow-up = 17,751 person-years), 405 (15.8%) died after a median of 4.7 (IQR = 3.4) years. Among the deceased, 148 (36.5%) died from CVD, 127 (31.4%) died from cancer, and 130 (32.1%) died from other causes of death. The most frequent single ICD-10 codes within CVD were atherosclerotic heart disease (ICD-10: I251, *n* = 29), acute myocardial infarction (ICD-10: I219, *n* = 21), and stroke (ICD-10: I64, *n* = 11). For cancer deaths, these were lung cancer (ICD-10: C349 *n* = 17), prostate cancer (ICD-10: C61, *n* = 13), and pancreas cancer (ICD-10: C259, *n* = 12). The largest groupings within other causes of death were 23 individuals who died from endocrine, nutritional, and metabolic diseases (ICD-10: E00-E99), 20 cases who died from respiratory diseases (ICD-10: J00-J99), 15 persons who died from infectious diseases (ICD-10: A00-B99, U071, including 10 COVID-19 deaths), and 16 deaths due to external causes. Mean age at death was lowest for cancer deaths (78.0, SD = 6.2 years), followed by other causes of death (80.6, SD = 7.6 years), and highest among participants who died from CVDs (82.4, SD = 7.4 years).

Figure 1 (plot D) and Table 2 indicate that survivors had lower median FI at baseline (0.11, IQR = 0.11) compared to those who died during follow-up (0.16, IQR = 0.15). The level of frailty also varied across different causes of death: older adults who died from cancer had a lower average FI (median = 0.13, IQR = 0.12) compared to CVD deaths (0.18, IQR = 0.16) and other causes of death (0.18, IQR = 0.17). Among the three most common ICD-10 codes within CVD mortality, the median FI was somewhat higher among those who died from atherosclerotic heart disease (0.21, IQR = 0.16) and acute myocardial infarction (0.20, IQR = 0.18) compared to those who died from stroke (0.16, IQR = 0.13). Among those who died from cancer, older adults with lung cancer were more frail (0.15, IQR = 0.07) and those with pancreatic cancer least frail (0.09, IQR = 0.09). Within other causes of death, FI levels were highest among those who later died from respiratory disease (0.23, IQR = 0.16), lower among older adults who died from endocrinological/nutritional/metabolic diseases (0.15, IQR = 0.22) or infectious diseases (0.14, IQR = 0.14), and lowest among those with external causes of death (0.10, IQR = 0.13).

**Fig. 2:**
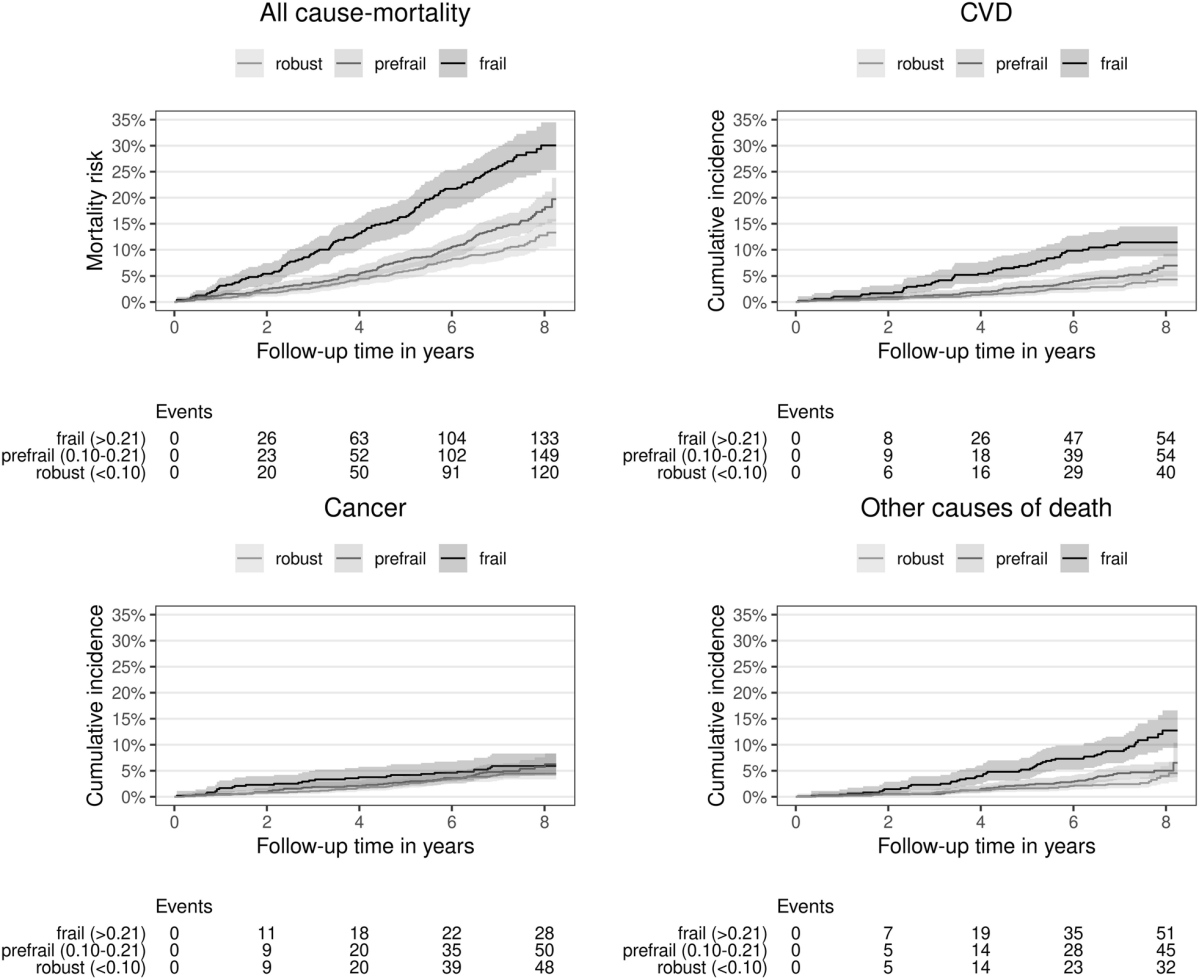
Unadjusted survival curves: Association between frailty index categories and all-cause and cause-specific mortality

Unadjusted survival curves between FI categories and all-cause and cause-specific mortality over time are depicted as Kaplan–Meier and cumulative incidence curves (Fig. 2). These indicate a higher risk of frail compared to robust older adults with regard to all-cause mortality, CVD mortality, and death from other causes. Finally, adjusted hazard ratios (HR) from Cox regression models for both the continuous and the categorized FI are provided in Table 3. Here, we find that the FI predicts both all-cause and cause-specific mortality, albeit to a varying degree. Adjusted all-cause mortality risk increased by 33% (HR = 1.33) for every 0.1 FI difference, and both pre-frail (HR = 1.30) and frail (HR = 2.40) older adults were at a higher risk to die during follow-up compared to robust older adults. Risk of death from CVDs according to FI levels and categories was similar to all-cause mortality, but lower for cancer (HR = 1.19 per 0.1 FI; HR = 1.47 for frail versus robust) and higher for other causes of death (HR = 1.53 per 0.1 FI, HR = 3.59 for frail versus robust). The 95%-confidence intervals of the HR of pre-frailty for cause-specific mortality risk overlapped with 1 (statistically non-significant). Sensitivity analysis (Supplementary Table 1) indicated that the FI predicted CVD mortality equally well when CVD diseases and risk factors were omitted from the FI calculation. Interaction effects of the FI with sex and level of education were tested (not shown), but the effects sizes were either small and/or statistically non-significant, and did not improve overall model fit. AUC values were acceptable for overall mortality (0.72), CVD (0.78) mortality and other causes of death (0.74), and poor for cancer mortality (0.64).

**Table 3:** Adjusted hazard ratios relating cause of death to continuous and categorized frailty index values

	Continuous FI (per 0.1)		Categorized FI		
			Prefrail	Frail	
Outcome	HR (95%-CI)	AUC (95%-CI)	HR (95%-CI)	HR (95%-CI)	AUC (95%-CI)
All-cause mortality	1.33 (1.22, 1.44)	0.722 (0.697, 0.747)	1.30 (1.02, 1.66)	2.40 (1.85, 3.10)	0.719 (0.694, 0.744)
CVD	1.25 (1.10, 1.42)	0.780 (0.741, 0.819)	1.33 (0.88, 2.00)	2.46 (1.60, 3.78)	0.784 (0.745, 0.823)
Cancer	1.19 (1.01, 1.40)	0.643 (0.592, 0.694)	1.19 (0.80, 1.77)	1.47 (0.91, 2.39)	0.636 (0.585, 0.686)
Other	1.52 (1.33, 1.74)	0.744 (0.699, 0.789)	1.47 (0.94, 2.30)	3.59 (2.27, 5.67)	0.737 (0.692, 0.780)

*N* = 2,561, unweighted data, Cox regression models adjusted for sex, living alone, and level of education. Reference category for the categorized FI is robust (FI < 0.1)

*HR* Hazard ratio, *95%-CI* 95% confidence interval, *AUC* Area under the curve refers to Harrell’s C index/concordance index, *CVD* Cardiovascular disease mortality

## Discussion

In this analysis, we found the FI to predict all-cause and cause-specific mortality during up to 8 years of follow-up in a population-representative sample of community-dwelling older adults in Austria. The FI was better at predicting mortality from CVDs and other causes of death compared to death from cancer. Finally, we found no evidence for a modification of the predictive power of the FI between men and women or across socio-economic strata (education).

Our study adds to the evidence summarized in systematic reviews and meta-analyses [8, 14, 25] which highlight the consistent association between the level of frailty and an increased overall mortality risk as well as the mostly acceptable discriminatory power of the FI [9]. The adjusted effects of the categorized FI show that both pre-frailty and frailty were statistically significantly associated with all-cause mortality, whereas the association was less consistent for cause-specific mortality with regard to pre-frailty, and with regard to frailty in case of cancer deaths. Our result that frailty predicted CVD mortality is in line with findings from previous studies assessing frailty for multiple causes of death [15, 17, 26] as well as with studies on CVD mortality [27, 28] or mortality among CVD patients [29]. As (pre-)frailty and (sub-clinical) CVD share a number of risk factors (e.g. smoking, poor diet, low socioeconomic position) and pathophysiological pathways (e.g. inflammation and oxidative stress markers) [30, 31], it is unclear whether the dose–response association between the frailty level – which describes a cumulative physiological decline [1] in older adults – and CVD mortality can be considered causal or is due to common causes. In our study, excluding CVD-related deficits from the FI (from 41 to 37 health deficits) at least did not alter the predictive capacity of the FI with regard to CVD mortality (Supplementary Table 1). Finally, it has also been hypothesized that causality between CVD and frailty may run both ways [32].

Our result that the continuous FI also predicts cancer deaths, albeit less so than CVD or mortality from other causes, differ from the results of two Swedish cohort studies [15, 16] which did not find an association, but are in line with more recent work based on other cohort studies from China [17], Germany [18], and Canada [19] as well as with studies which provide evidence that frailty predicts mortality among cancer patients [33] and cancer survivors [34]. The nature of the association between frailty and cancer again remains ambiguous: both are strongly age-related and share a number of risk factors (e.g. lifestyle factors [30, 35]) which might account for the association between cancer mortality and frailty. But, cancer and cancer treatments can deplete physiological reserves and may hence also cause frailty [36], just as well as frailty can be considered a risk factor for cancer incidence [37]. That the association between frailty and cancer mortality is smaller and less consistent compared to CVD and other mortality could be due to low survival rates of certain cancers, e.g. pancreatic, liver or lung cancer, which often prove lethal even in robust older adults. Importantly, it has been shown that a frailty assessment followed by frailty interventions can increase both the treatment tolerability and feasibility in older patients receiving systematic cancer therapy [38].

Finally, unlike Jiang et al. [15], but in accordance with other studies [17, 26] we found no evidence for differences in the predictive power of the FI between men and women. We also found no difference in the effect of the FI on mortality risk across socioeconomic status (education). This suggests that the FI can be used as a means for risk assessment across socio-demographic groups.

Strengths of our study stem mostly from the study design: a large sample representative of the community-dwelling older population in Austria was followed up to 8 years, (the level of) frailty comprehensively assessed by use of a frailty index based on 41 health deficits, and high-quality official cause of death statistics were available. Our study also has three important limitations. First, since only 15% (*n* = 405) of the survey participants aged 65 + had died during follow-up, causes of death had to be categorized fairly broadly (CVD/cancer/other). There were too few deaths from within these categories, for example with regard to respiratory, and infectious diseases (including COVID-19) to be analysed. Second, the FI created from the Austrian Health Information Survey relied exclusively on self-reported information, which may have led to an underestimation of frailty levels [39]. Third and finally, frailty was assessed only at baseline, although the frailty level of the survey participants likely increased during the 8 years of follow-up. Previous studies suggest that FI increases are relevant for mortality prediction [10–12] and that using only single-time-point assessments results in an ever-decreasing predictive capacity of the FI [18]. In other words, risk stratification based on the FI would be more accurate and potentially useful if repeated assessments were available, for example, if frailty screening were conducted regularly during annual medical check-ups.

In conclusion, the FI predicts all-cause and cause-specific mortality, which points towards the importance of frailty screening as a means for risk stratification.

### Supplementary Information

**Additional file 1: Supplementary Table 1.** Adjusted hazard ratios relating cause of death to continuous and categorized frailty index values (without CVD diagnoses or risk factors).

## Data Availability

ATHIS 2014 data can be obtained from Statistics Austria free of charge upon reasonable request (info@statistik.gv.at) and individual-level linkage of ATHIS 2014 survey data with cause of death information can be requested via the Austrian Micro Data Center (AMDC: https://www.statistik.at/services/tools/services/center-wissenschaft/austrian-micro-data-center-amdc) as has been done for the current paper (AMDC project C0N63). Access to data from the Austrian Micro Data Center (AMDC) for a researcher is provided on a project basis, and requires the accreditation of the scientific host institution, submitting an online application, and signing of a contract between the scientific host institution and the AMDC. More information can be found here (in German): https://www.statistik.at/services/tools/services/center-wissenschaft/austrian-micro-data-center-amdc. Information on the AMCD data catalogue is available here: https://www.statistik.at/amdc-data/#/product. More information specifically on ATHIS 2014 (in German) is available at https://www.statistik.at/en/services/tools/services/publikationen/detail/1074 (accessed 18th September 2023).
