# Supplementary material for: Frailty predicts all-cause and cause-specific mortality among older adults in Austria: 8-year mortality follow-up of the Austrian Health Interview Survey (ATHIS 2014)
Source: BMC Geriatr. 2024 Jan 3;24:13. doi: 10.1186/s12877-023-04633-3 (PMC10765716; doi:10.1186/s12877-023-04633-3)
Supplement: Supplementary file 1 — Additional file 1: Supplementary Table 1. Adjusted hazard ratios relating cause of death to continuous and categorized frailty index values (without CVD diagnoses or risk factors). [file 12877_2023_4633_MOESM1_ESM.docx]

Supplementary Table 1: Adjusted hazard ratios relating cause of death to continuous and categorized frailty index values (without CVD diagnoses or risk factors)

|  | Continuous FI (per 0.1) | | Categorized FI | | |
| --- | --- | --- | --- | --- | --- |
|  |  |  | Prefrail | Frail |  |
| Outcome | HR (95%-CI) | AUC (95%-CI) | HR (95%-CI) | HR (95%-CI) | AUC (95%-CI) |
| CVD mortality | 1.21 (1.07, 1.37) | 0.779 (0.740, 0.818) | 1.39 (0.93, 2.09) | 2.19 (1.44, 3.35) | 0.783 (0.744, 0.822) |

N = 2,561, unweighted data, Cox regression models adjusted for age, sex, living alone, and level of education. In contrast to the 41-item FI described above, we excluded three CVD diagnoses (heart infarction, chronic heart disease, and stroke) and one CVD risk factor (hypertension) for the calculation and hence used only the remaining 37 health deficits. Reference category for the categorized FI is robust (FI<0.1). HR = hazard ratio, 95%-CI = 95% confidence interval, AUC = area under the curve refers to Harrell’s C-Index/concordance index, CVD = cardiovascular disease.
